# Intra-patient comparison of physiologic ^68^Ga-PSMA-11 and ^18^F-DCFPyL PET/CT uptake in ganglia in prostate cancer patients: a pictorial essay

**DOI:** 10.1186/s40644-021-00404-8

**Published:** 2021-04-16

**Authors:** Medhat M. Osman, Amir Iravani, Michael S. Hofman, Rodney J. Hicks

**Affiliations:** 1 Division of Nuclear Medicine, Department of Radiology, Saint Louis University Hospital, St. Louis, MO USA; 2 Mallinckrodt Institute of Radiology, Washington University, St. Louis, MO USA; 3 Centre for Molecular Imaging, Department of Cancer Imaging, Peter MacCallum Cancer Centre, Melbourne, Victoria Australia; 4 Sir Peter MacCallum Department of Oncology, University of Melbourne, Melbourne, Victoria Australia

**Keywords:** ^68^Ga-PSMA11 PET/CT, ^18^F-DCFPyL PET/CT, PSMA, Ganglia

## Abstract

**Background:**

Recent studies reported metabolic uptake in at least one of the evaluated ganglia in 98.5% of patients undergoing ^68^Ga -PSMA-11 and in 96.9% of patients undergoing ^18^F-DCFPyL PET/CT examination. We have observed different patterns of ganglion visualization with ^18^F-DCFPyL compared to ^68^Ga-PSMA-11. This includes more frequent visualization of cervical and sacral ganglia, which may be attributable to better imaging characteristics with ^18^F PET imaging.

**Case presentation:**

This pictorial essay is to illustrate and compare, in the same patient, various representative cases of ^68^Ga-PSMA-11 and ^18^F-DCFPyL PET/CT uptake in ganglia at different anatomic locations, with different patterns and distribution of metabolic activity.

**Conclusion:**

Reading physicians should be aware of the frequently encountered and occasionally different physiologic uptake of ^68^Ga-PSMA-11 and ^18^F DCFPyL in different ganglia.

## Background

Several recent studies have revealed accurate primary staging and restaging after biochemical recurrence of prostate cancer (PC) using Gallium-68 Prostate Specific Membrane Antigen (^68^Ga -PSMA PET/CT) [1–3]. However, ^68^Ga -PSMA-11 uptake is not completely specific to PC [4–6]. Whilst experience initially evolved with ^68^Ga –PSMA-11 PET/CT, there is increasing use of ^18^F-labelled PSMA radiotracers, which may have advantages for large-scale production fulfilling good manufacturing practice (GMP). There is also the possibility that such agents may improve imaging quality owing to better nuclear decay characteristics including shorter positron emission range [7]. High ^68^Ga -PSMA-11 uptake was initially described in coeliac ganglia, where it may mimic lymph node metastases [8]. More recently, uptake in stellate ganglia has also been recognized on ^68^Ga -PSMA-HBED PET/CT imaging [9]. However, PSMA uptake in ganglia may also visualize additional ganglia within the imaged field of view. Recent studies reported metabolic uptake in at least one of the evaluated ganglia in 98.5% of patients undergoing ^68^Ga -PSMA and in 96.9% of patients undergoing 2-(3-(1-carboxy-5-[(6-[^18^F]fluoro-pyridine-3-carbonyl)-amino]-pentyl)-ureido)-pentanedioic acid (^18^F-DCFPyL) PET/CT examination [10, 11]. We have observed different patterns of ganglion visualization with ^18^F-DCFPyL compared to ^68^Ga-PSMA-11. This includes more frequent visualization of cervical and sacral ganglia, which may be attributable to better imaging characteristics with ^18^F PET imaging. Surprisingly, we also observed less frequent visualization of stellate and coeliac ganglia, which underscores the differences in chemical bio distribution between ^68^Ga -PSMA-11 and ^18^F -DCFPyL radiotracers. Furthermore, it has been recently demonstrated that the use of modern PET/CT employing time-of-flight information is leading to higher number of visualizations of uptake in physiological structures such as ganglia [12]. Therefore, PSMA uptake in any ganglia may represent a common potential pitfall in staging and restaging patients with PC undergoing ^68^Ga-PSMA-11 or ^18^F -PSMA PET/CT examination. Most recently, the higher special resolution of ^18^F -PSMA-1007 PET/CT compared to ^68^Ga-PSMA-11 resulted in higher frequency of detecting non-tumor-related uptake in ganglia; however, comparison was limited to matched-pair cohorts of patient [13]. To date, no studies directly comparing different PSMA-ligand ganglia uptake in the same patient are available. Of importance, new drug application (NDA) for both ^68^Ga-PSMA-11 or ^18^F - DCFPyL PET/CT has been submitted to Food and Drug Administration (FDA) and these two will likely be first agents entering into clinical practice in the USA. However, in Europe a variety of other high affinity agents are being used. With potential FDA approval of both PSMA agents, the interpreting physician needs to be familiar with this important pitfall for reporting these studies. This manuscript aims to demonstrate the variation in intensity of uptake in ganglia in different anatomical regions, which should not be mistaken with development or disappearance of disease if patients were imaged sequentially with different PSMA agents during the course of the disease.

This pictorial essay is to illustrate and compare various representative cases from prostate cancer patients who had both ^68^Ga-PSMA-11 and ^18^F-DCFPyL PET/CT that revealed different patterns of PSMA uptake. The case studies include unilateral and bilateral uptake, mild and moderate uptake, focal and diffuse uptake and emphasize the importance of careful correlation with the CT scan as part of the ^68^Ga-PSMA-11 and ^18^F-DCFPyL PET/CT examination in differentiating physiologic uptake in ganglia versus lymph nodes. Anatomic location and morphology by CT and the generally mild ^68^Ga-PSMA-11 or ^18^F-DCFPyL uptake should help the interpreting physician distinguish physiological uptake within ganglia from pathological uptake in lymph nodes. Associated CT findings and characterization of ganglia in the ^68^Ga-PSMA-11 and ^18^F-DCFPyL PET/CT will be demonstrated at different locations including cervical, stellate, coeliac, lumbar and sacral regions.

## Case presentations

Cases were selected from a group of prostate cancer patients who had both ^68^Ga-PSMA-11 and ^18^F-DCFPyL. In all patients ^68^Ga-PSMA-11 was done first, with a median time interval between scans of 22.5 months. Selected caseshad small to moderate tumor load (PSA < 20 ng/mL and interval PSA change < 10 ng/ml between the two studies), to avoid impact of potential tumor steal in patients with high tumor burden. It is assumed that, as normal structures, physiological activity in ganglia is unlikely to change significantly over time.

### ^68^Ga -PSMA-11 PET/CT image acquisition and protocol

The CT portion of the study was performed in a cranio-caudal direction encompassing vertex to mid-thigh using the following parameters, slice thickness of 3.25 mm, increments of 1.5 mm, 140 keV, 220 mAs and 0.6 pitch. This was performed 10 min (range 8–15) following intravenous injection of 50mls of Omnipaque 300 g/ml contrast medium (GE Healthcare, Princeton, NJ) for optimal ureteric enhancement. We have recently described the advantages of this CT Urography (CT-U) protocol on differentiating nodes from focal pooling of urine [14]. Renal function was assessed by estimated glomerular filtration rate (eGFR) on peripheral blood test prior to contrast injection. Prior history of allergic reaction to intravenous contrast was sought. PET images were acquired approximately 62 min (range 40–85) following injection of 2 MBq/kg of ^68^Ga PSMA-labeled Glu-NH-CO-NH-Lys-(Ahx) HBED (166 MBq, range 91–246). This was timed immediately after CT scanning in the supine position on the same integrated PET/CT camera from vertex to mid-thigh in a 3-D (matrix 168 × 168) mode using GE Discovery PET/CT 690 (GE Healthcare, Milwaukee, WI), GE Discovery PET/CT 710 (GE Healthcare, Milwaukee, WI), and Siemens Biograph 16 PET/CT (Siemens Healthcare, Erlangen, Germany). The emission data was corrected for random, scatter and decay. Reconstruction was conducted with an ordered subset expectation maximization (OSEM) algorithm with 2 iterations/8 subsets and Gauss-filtered to a transaxial resolution of 5 mm at full-width at half-maximum (FWHM). Attenuation correction was performed using above mentioned CT-U data. PET and CT were performed using the same protocol for every patient on all cameras.

### ^18^F-DCFPyL PET/CT image acquisition and protocol

The CT portion of the study is identical to that of ^68^Ga-PSMA scan. PET images were acquired approximately 120 min following injection of weight based 3.57 Megabecquerel/Kilogram MBq/kg. This was timed immediately after CT scanning in the supine position on the same integrated PET/CT camera from vertex to mid-thigh in a 3-D (matrix 168 × 168) mode using GE Discovery PET/CT 690 (GE Healthcare, Milwaukee, WI), GE Discovery PET/CT 710 (GE Healthcare, Milwaukee, WI). The emission data was corrected for random, scatter and decay. Reconstruction was conducted with an ordered subset expectation maximization (OSEM) algorithm with 2 iterations/18 subsets and Gauss-filtered to a transaxial resolution of 5 mm at FWHM. Attenuation correction was performed using above mentioned CT-U data. PET and CT were performed using the same protocol for every patient on all cameras.

### Image analysis

For each patient who had both ^68^Ga -PSMA-11 and ^18^F –DCFPyL scans, imaging data were analyzed and case studies were captured using MIM Encore (MIM Software Inc., Cleveland, Ohio, USA). Each figure is for a patient who had both ^68^Ga -PSMA-11 and ^18^F –DCFPyL and images were captured at the same level and projection in order to compare uptake with both radiotracers at the same location.

### Cervicothoracic region

**Fig. 1:**
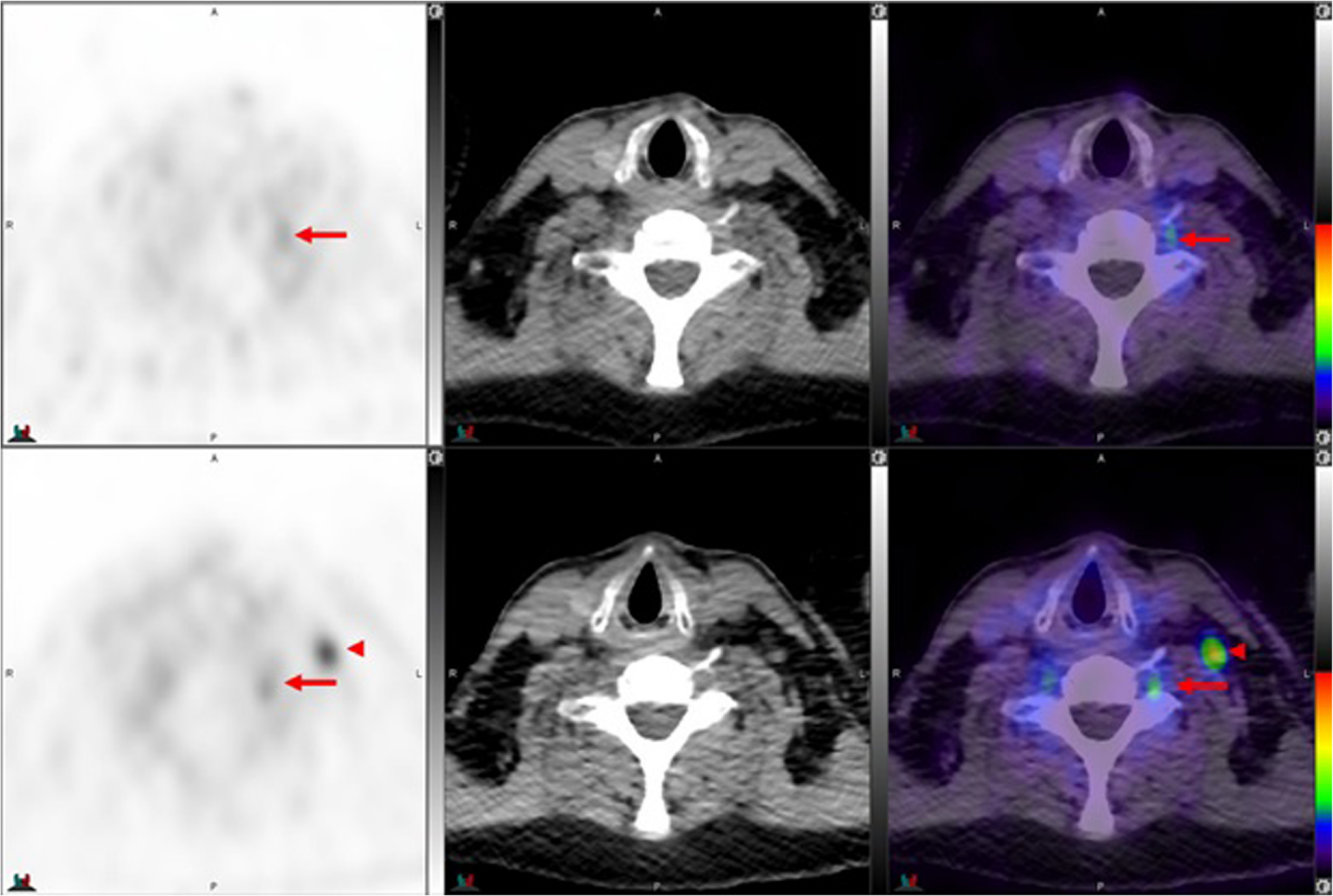
Top panel ^68^Ga-PSMA-11: PET, CT and PET/CT. Left cervical ganglion (arrow) maximum Standard Uptake Value (SUVmax) = 2.2. Lower panel ^18^F –DCFPyL: PET, CT and PET/CT. Left cervical ganglion (arrow) SUVmax = 2.6, Right cervical ganglion SUVmax = 2.3. Of note, ^18^F –DCFPyL shows new left supraclavicular metastases (arrow head). The color intensity has been slightly adjusted to highlight the uptake in the ganglia. There is higher and bilateral lower cervical ganglia uptake with ^18^F –DCFPyL. By CT, there is typically no anatomic structure detectable in the region of cervical ganglia; however, location, bilaterality and multiplicity would provide clues

**Fig. 2:**
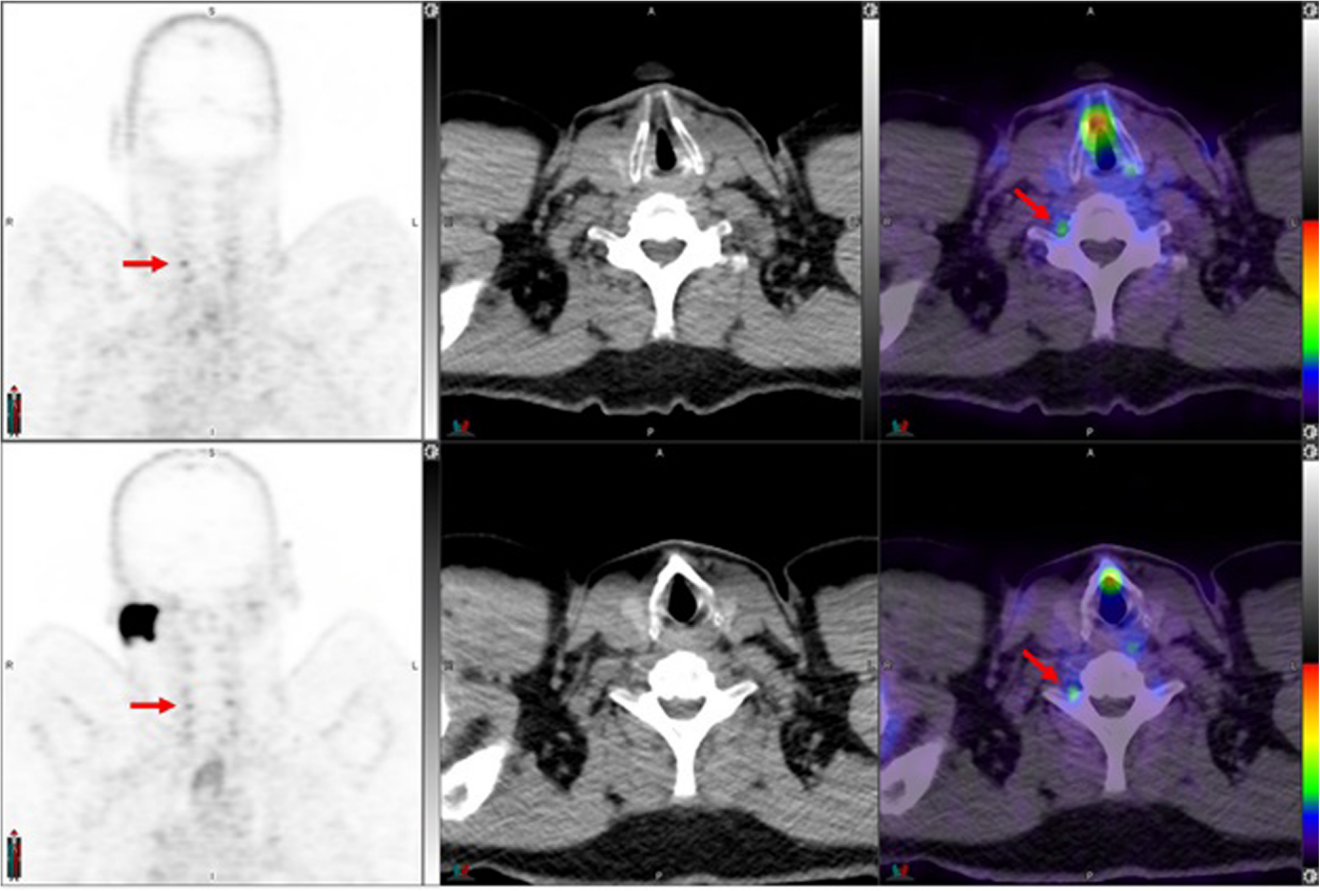
Top panel ^68^Ga-PSMA-11: coronal PET, axial CT and axial fused PET/CT. Right cervical ganglion (arrow) SUVmax = 2.4. Lower panel ^18^F –DCFPyL: PET, CT and PET/CT. Right cervical ganglion (arrow) SUVmax = 2.5. The color intensity has been slightly adjusted to highlight the uptake in the ganglia. Higher and bilateral uptake in lower cervical ganglia noted with ^18^F –DCFPyL

**Fig. 3:**
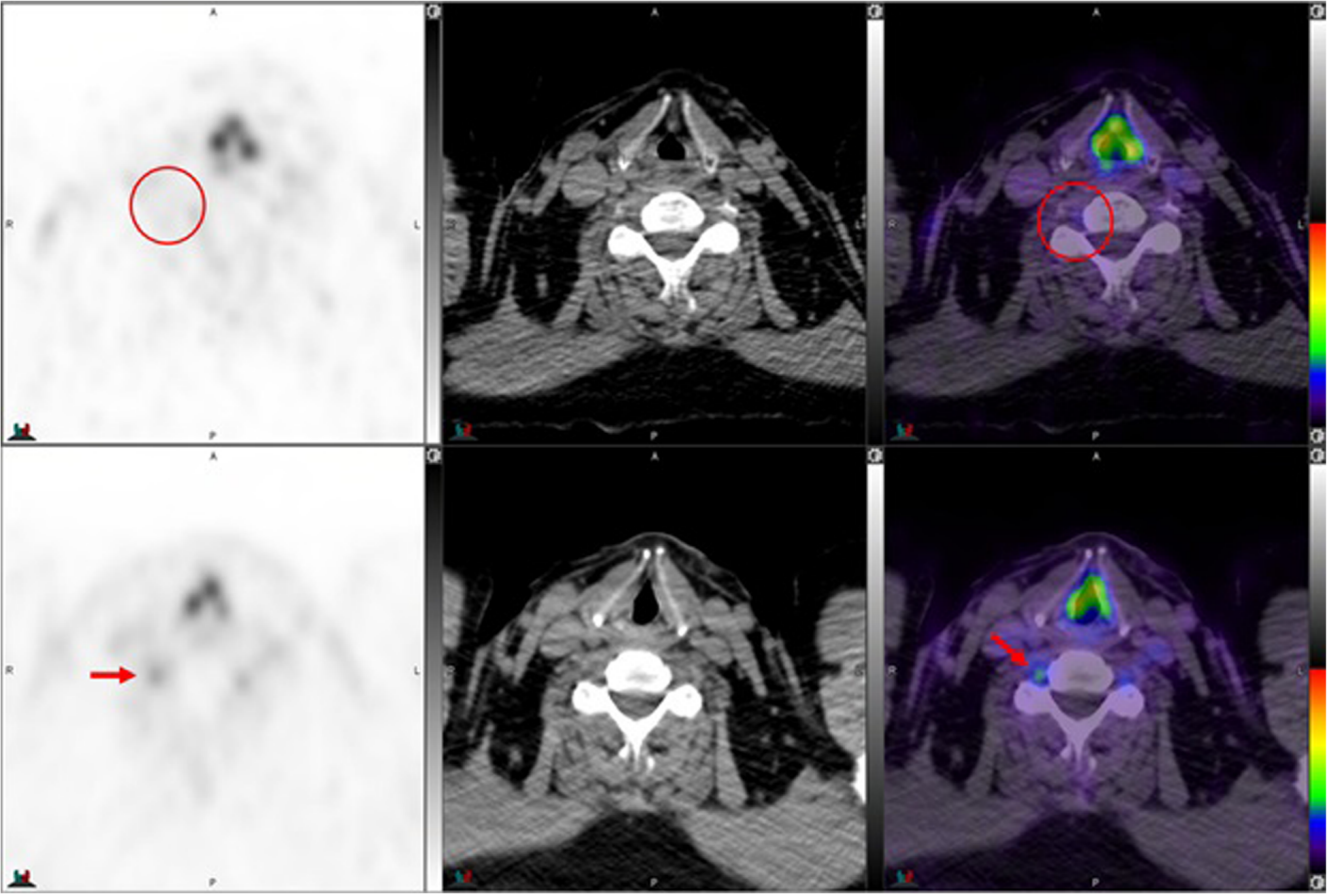
Top panel ^68^Ga-PSMA-11: axial PET, axial CT and axial fused PET/CT. Region of the Right lower cervical ganglion (circle). Lower panel ^18^F –DCFPyL: PET, CT and PET/CT. Right lower cervical ganglion uptake (SUVmax = 2.5) noted with ^18^F –DCFPyL (arrow)

**Fig. 4:**
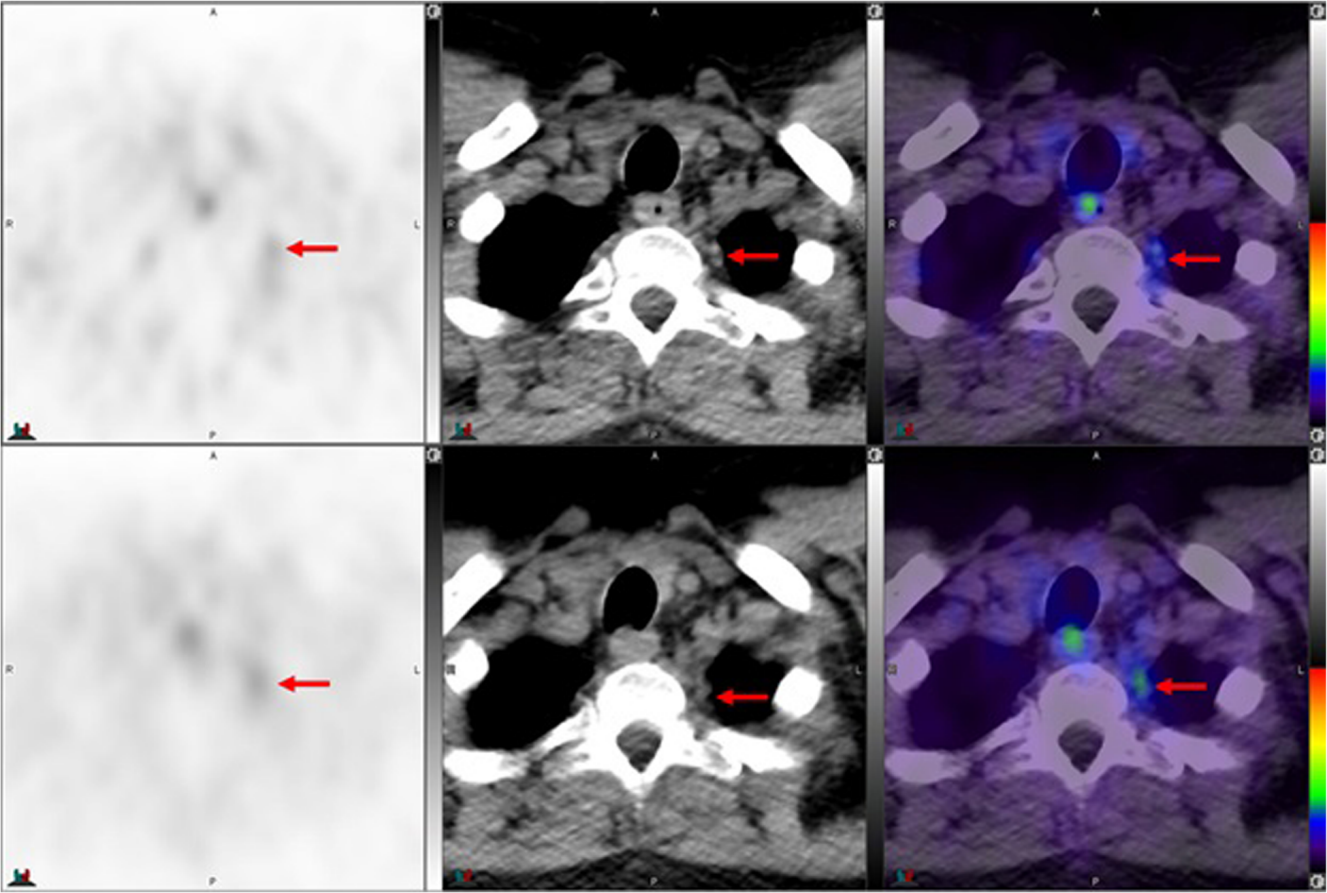
Top panel ^68^Ga-PSMA-11: axial PET, axial CT and axial fused PET/CT. Lower panel ^18^F –DCFPyL: axial PET, axial CT and axial fused PET/CT. The color intensity has been slightly adjusted to highlight the uptake in the ganglia (arrows). Left stellate ganglion with ^18^F –DCFPyL (SUVmax = 2.5) > ^68^Ga-PSMA-11 (SUVmax = 2.1). Metabolic activity is more commonly noted in the left side. By CT, stellate ganglia are typically located anterior to first rib and have curvilinear or nodular shape

**Fig. 5:**
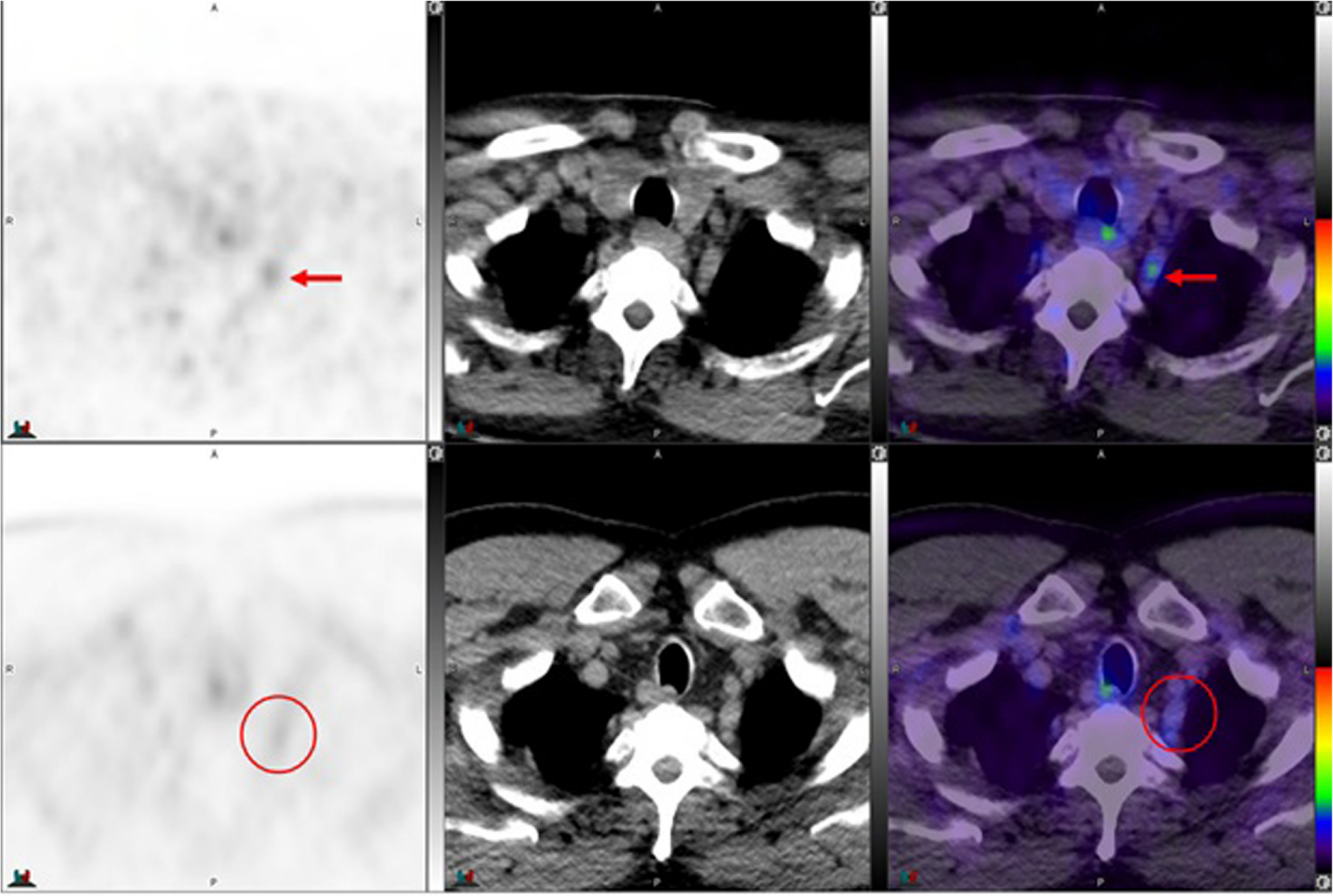
Top panel ^68^Ga-PSMA-11: axial PET, axial CT and axial fused PET/CT. Lower panel ^18^F –DCFPyL: axial PET, axial CT and axial used PET/CT. The color intensity has been slightly adjusted to highlight the uptake in the ganglia (arrows in the top panel and circles in the lower panel). Left stellate ganglion uptake with ^68^Ga-PSMA-11 only (SUVmax = 1.8)

**Fig. 6:**
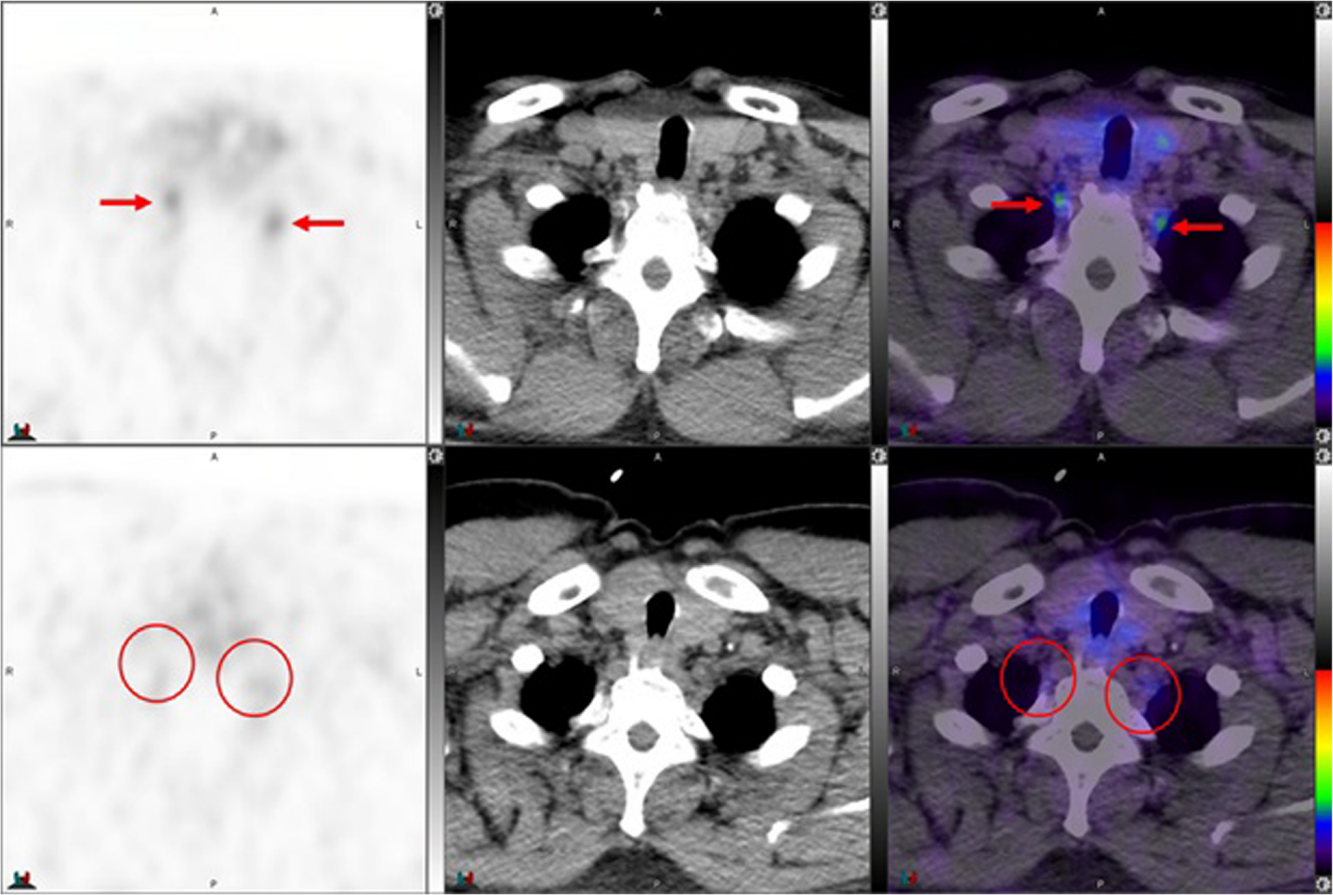
Top panel ^68^Ga-PSMA-11: axial PET, axial CT and axial fused PET/CT. Lower panel ^18^F –DCFPyL: axial PET, axial CT and axial fused PET/CT. The color intensity has been slightly adjusted to highlight the uptake in the ganglia (arrows in the top panel and circles in the lower panel). Bilateral stellate ganglia uptake with ^68^Ga-PSMA-11 (left and right stellate ganglia SUVmax = 2.7 and 3.0, respectively) but not with ^18^F –DCFPyL

Cervical ganglia innervating the head and neck regions, comprise three paravertebral ganglia; superior, middle and inferior cervical ganglia. All are parts of the sympathetic nervous system that play major roles in regulating the autonomic nervous system (ANS). In approximately 80% of the population, the inferior cervical ganglion fuses with the first thoracic ganglion, forming the stellate (cervicothoracic) ganglion [15]. Stellate ganglia are the sites of blockade for several sympathetic hyperactivity-associated disorders including cardiac arrhythmia, pain syndromes and even post-traumatic stress disorder (PTSD) [16–18]. It is not clear if the detection or the intensity of Ga-PSMA-11 uptake in stellate ganglia have any clinical significance. Anatomic localization of the stellate ganglia is critical in the differentiation of metabolically active ganglia from a pathologic lymph node. Stellate ganglia are typically located at the transverse process of C6- C7, inferior to subclavian artery, and superior/anterior to the neck of the first rib. However, there could be anatomic variations secondary to neck extension and neutral supine positions [18]. Size (~ 10 × 20 mm) and metabolic activity are likely reasons why PSMA uptake in stellate ganglia is more noticeable than any other ganglia in the cervicaothoracic region. Uptake in at least one stellate ganglion have been noted in 80% of studied prostate cancer cases undergoing ^68^Ga-PSMA-11 examination [9]. More recently, a study reported cervical (including stellate) ^68^Ga-PSMA- HBED uptake in 92% of patients [10]. In comparison, discernible ^18^F-DCFPyL uptake in cervical and stellate ganglia was noted in 67.1% and of 65.8% of prostate cancer cases, respectively, in another study [11]. Since it would be expected that there would be relatively more frequent visualization of uptake in cervical ganglia with ^18^F-DCFPyL PET/CT than with ^68^Ga-PSMA-11 due to the inherently higher spatial and contrast resolution, the lower incidence of visualization may plausibly reflect differences in tissue selectivity of these tracers [19]. Additionally, differences in injected doses and acquisition parameters may also contribute to ganglia uptake discrepancies. In our experience, some patients showed more frequent visualization of cervical ganglion with^18^F-DCFPyL compared to ^68^Ga-PSMA-11. Such uptake is easily recognizable based on anatomic location, configuration/shape, bilaterality and multiplicity. Conversely, we noted less frequent and lower SUVmax with^18^F-DCFPyL compared to ^68^Ga-PSMA-11 in the stellate ganglia. Representative cases of cervical and stellate uptake detected with ^68^Ga-PSMA-11 and ^18^F -DCFPyL radiotracers in same patients are shown in Figs. 1, 2, 3, 4, 5 and 6.

### Abdominal region

#### Coeliac ganglia

**Fig. 7:**
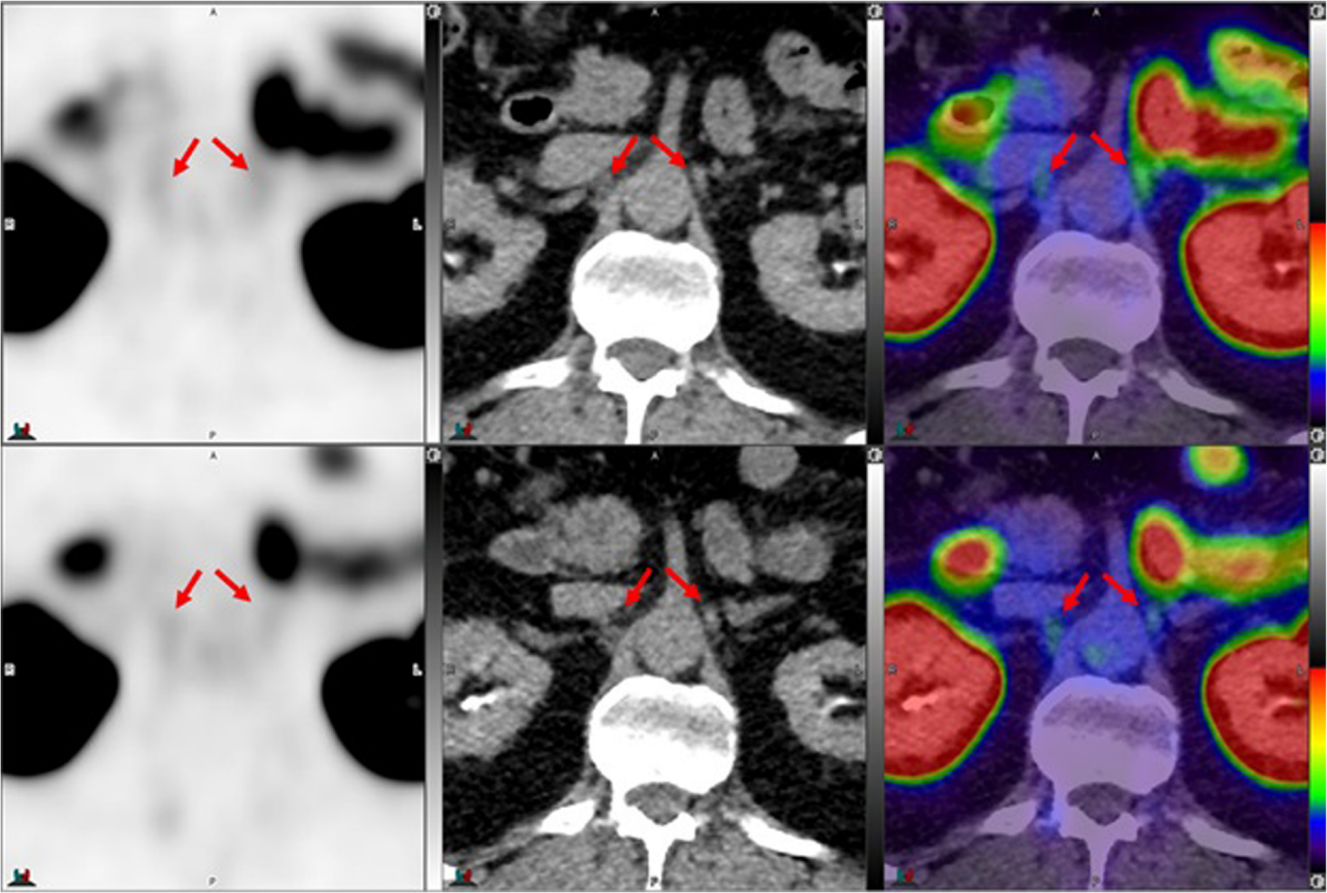
Top panel ^68^Ga-PSMA-11: axial PET, axial CT and axial fused PET/CT. Lower panel ^18^F –DCFPyL: axial PET, axial CT and axial fused PET/CT. Left coeliac ganglion uptake with ^68^Ga-PSMA-11 (SUVmax 2.4) > ^18^F –DCFPyL (SUVmax 2.2) (left arrows). Right coeliac ganglion shows similar uptake with both tracers (SUVmax = 2.1) (right arrows). By CT, coeliac ganglia are typically located adjacent to coeliac axis and have discoid, band or teardrop shape

**Fig. 8:**
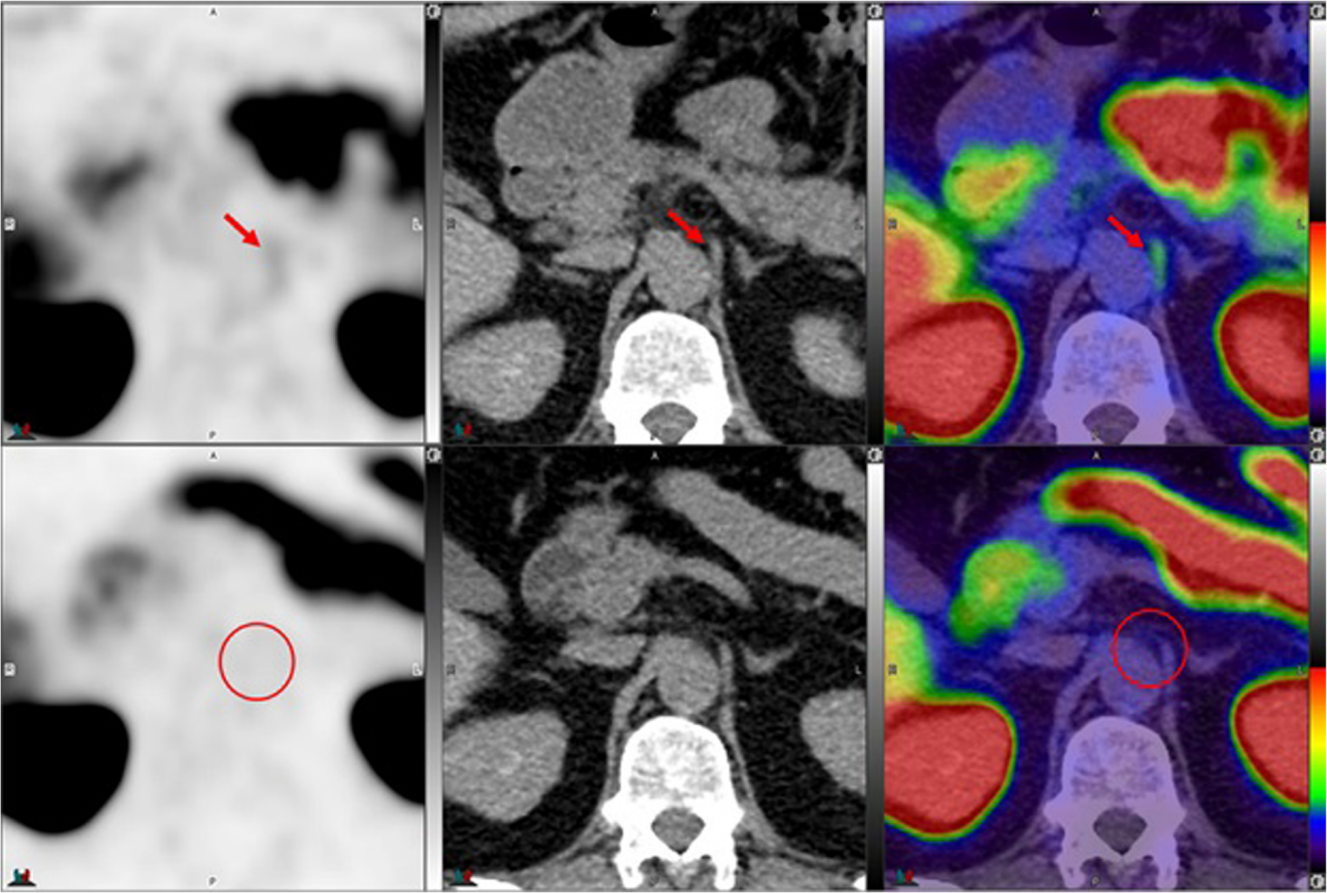
Top panel ^68^Ga-PSMA-11: axial PET, axial CT and axial fused PET/CT. Lower panel ^18^F –DCFPyL: axial PET, axial CT and axial fused PET/CT. The color intensity has been slightly adjusted to highlight the uptake in the ganglia. Left coeliac ganglion uptake with ^68^Ga-PSMA-11 (SUVmax = 2.7) (arrows) but not with ^18^F –DCFPyL (circles)

**Fig. 9:**
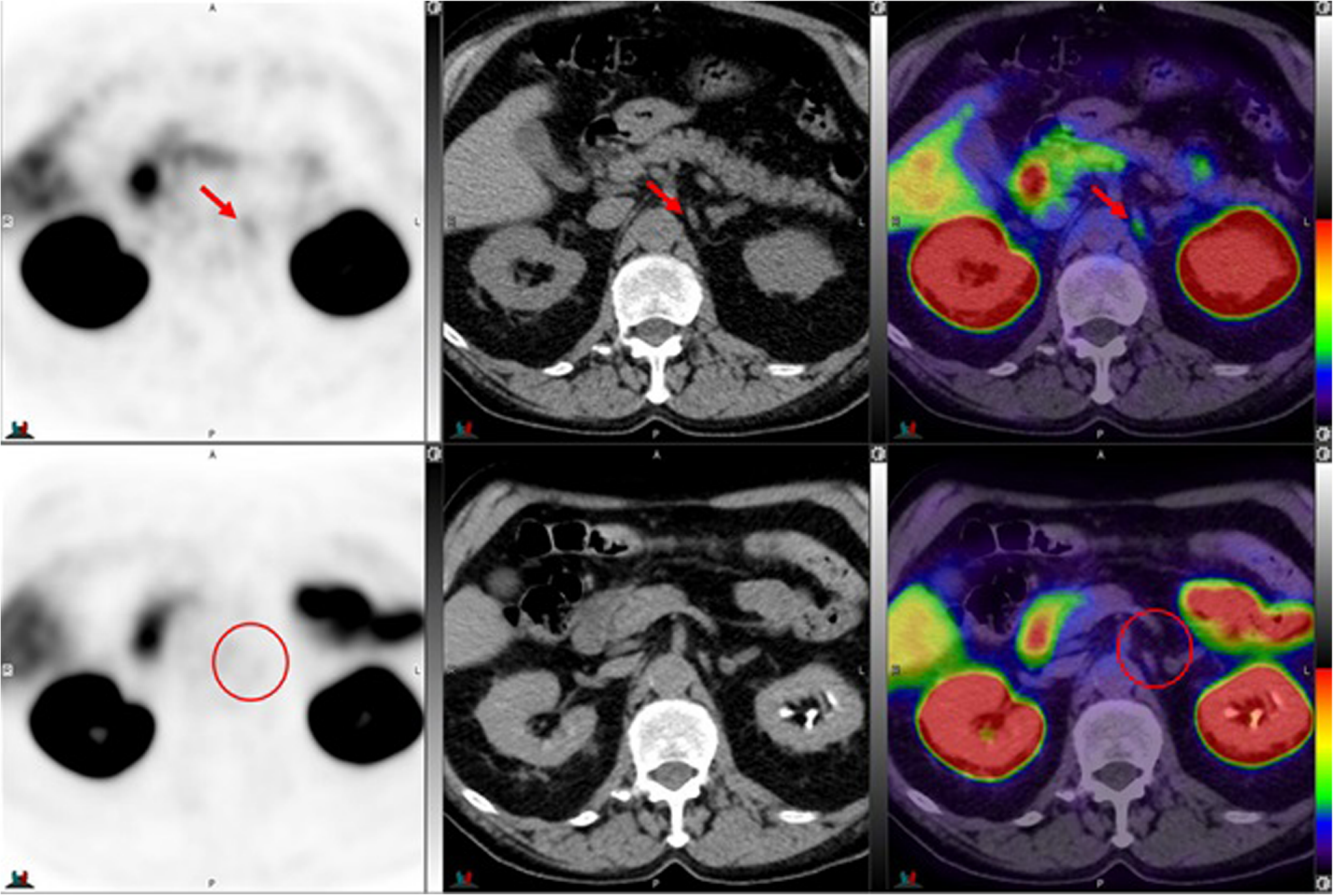
Top panel ^68^Ga-PSMA-11: axial PET, axial CT and axial fused PET/CT. Lower panel ^18^F –DCFPyL: axial PET, axial CT and axial fused PET/CT. Left coeliac ganglion uptake with ^68^Ga-PSMA (SUVmax = 2.4) (arrows) but not with ^18^F –DCFPyL (circles). By CT, the left coeliac ganglion is typically located between the coeliac trunk and left adrenal gland

Part of the sympathetic subdivision of the ANS, the two coeliac ganglia innervate the stomach, liver, pancreas, and duodenum. They can be identified based on their characteristic locations at the level between the origins of coeliac and superior mesenteric arteries and anterior to the diaphragmatic crura. The coeliac are the largest ganglia in the ANS; therefore, they are the most frequently seen ganglia in radiology. Using endoscopic ultrasonography, coeliac ganglia were identified in 51 out of 57 patients (89.4%) [20]. Coeliac ganglia can also be recognized based on CT morphological characteristics at the root of the superior mesenteric artery; its density is the same as the liver and spleen or slightly less than the diaphragmatic crura. The ganglia are detectable in most patients and not round like a lymph node but rather band-shaped, multilobulated, discoid or teardrop configuration. In one study using MDCT, coeliac ganglia are detectable in most patients, and the left coeliac ganglion was larger and visualized more often than the right ganglion (89% vs 67%, *P* < 0.0001) [21]. That is probably why if one coeliac ganglion shows detectable PSMA-avidity it is more likely to be the left side. The incidence of detecting metabolically active coeliac ganglia with FDG PET/CT has not been reported. Of importance, physiologic PSMA uptake in coeliac ganglia is a common potential false positive in PC patients. Notwithstanding misregistration artifacts, most metabolically active cervical, stellate, paravertebral lumbar and sacral ganglia can be distinguished anatomically by the CT portion the PET/CT from metastatic lymph nodes. Such differentiation is, however, potentially more challenging in the case of coeliac ganglia due to its location in the upper abdomen and the significant interpatient anatomic variability. In most cases, the degree of PSMA uptake in coeliac ganglia is typically less intense than in lymph node metastases from PC. In addition to location, the non-spherical shape may provide clues. In one study, physiologic PSMA uptake in coeliac ganglia was detectable in 89.4% of PC patients undergoing ^68^Ga-PSMA-11 PET/CT examination [8]. In the same study, histochemical analysis confirmed strong expression of PSMA in the nerve cells within the coeliac ganglia. A more recent study reported 84% of patients having ^68^Ga -PSMA-11 PET/CT uptake in at least one coeliac ganglion [9]. Both studies reported that bilateral ^68^Ga- PSMA-11 uptake was noted in 42–49% of studied patients and more frequent uptake in the left relative to the right side. A similar rate of bilaterality and left predominance has been reported ^68^Ga- PSMA-11 uptake in the stellate ganglia [10]. In comparison, Werner et al. reported no laterality preference and only 59.2% coeliac uptake in prostate cancer patients undergoing ^18^F -DCFPyL scans [11]. Similar to stellate ganglia, lower detection rate in ^18^F -DCFPyL coeliac uptake, despite the higher image resolution, underscores the differences in chemical biodistribution between ^68^Ga -PSMA-11 and ^18^F -DCFPyL radiotracers. Representative cases of coeliac uptake detected with ^68^Ga -PSMA-11 and ^18^F -DCFPyL radiotracers in same patients are shown in Figs. 7, 8 and 9.

### Lower pelvic region

#### Lumbar and sacral ganglia

**Fig. 10:**
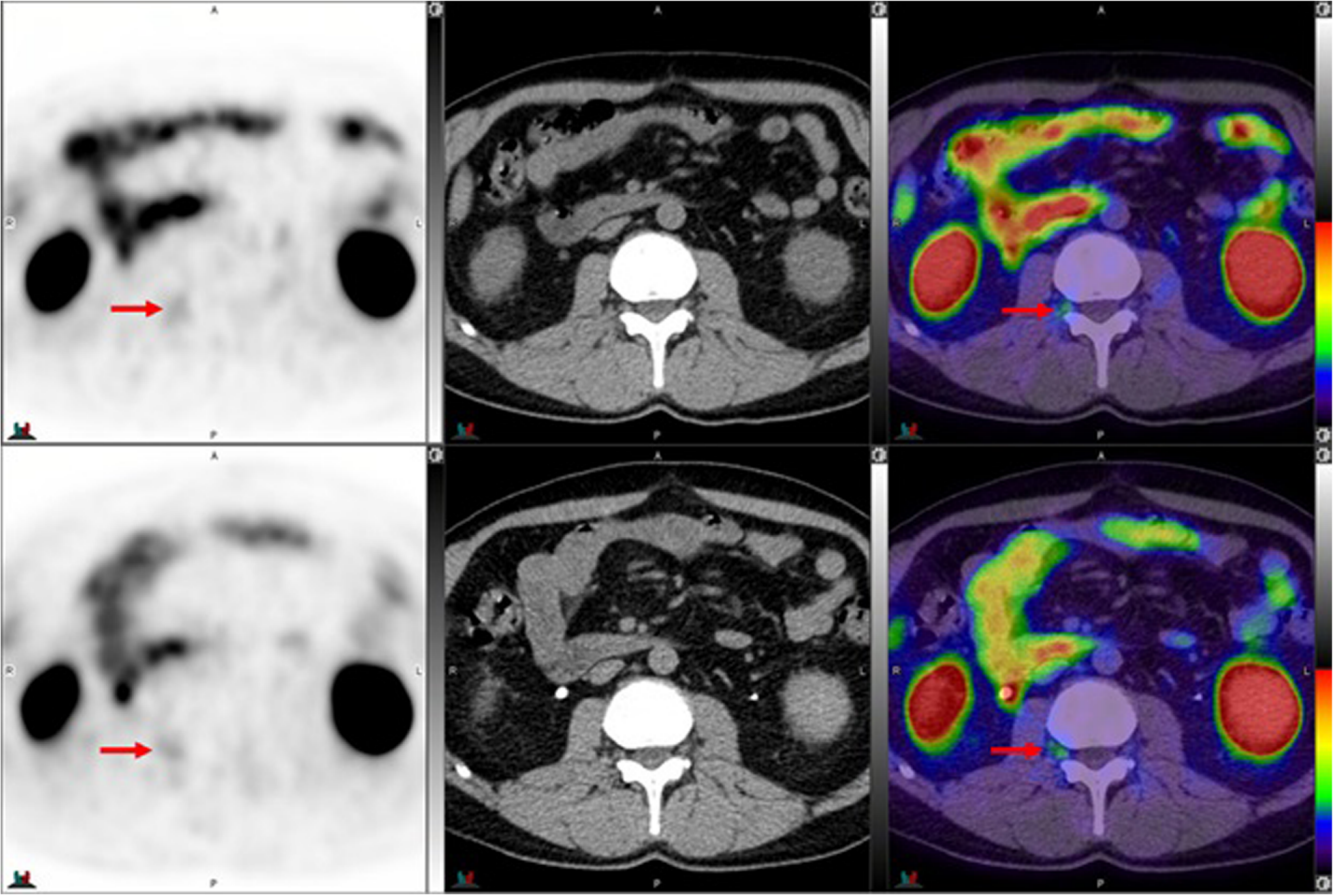
Top panel ^68^Ga-PSMA-11: axial PET, axial CT and axial fused PET/CT. Lower panel ^18^F –DCFPyL: axial PET, axial CT and axial fused PET/CT. The color intensity has been slightly adjusted to highlight the uptake in the ganglia. Right upper lumbar ganglion (arrows) uptake with ^18^F –DCFPyL (SUVmax=2.4) > with ^68^Ga-PSMA-11 (SUVmax=2.3). By CT, similar to cervical ganglia, there are typically no anatomic structures detectable in the region of lumbar ganglia; however, paravertebral location, bilaterality and multiplicity would provide clues

**Fig. 11:**
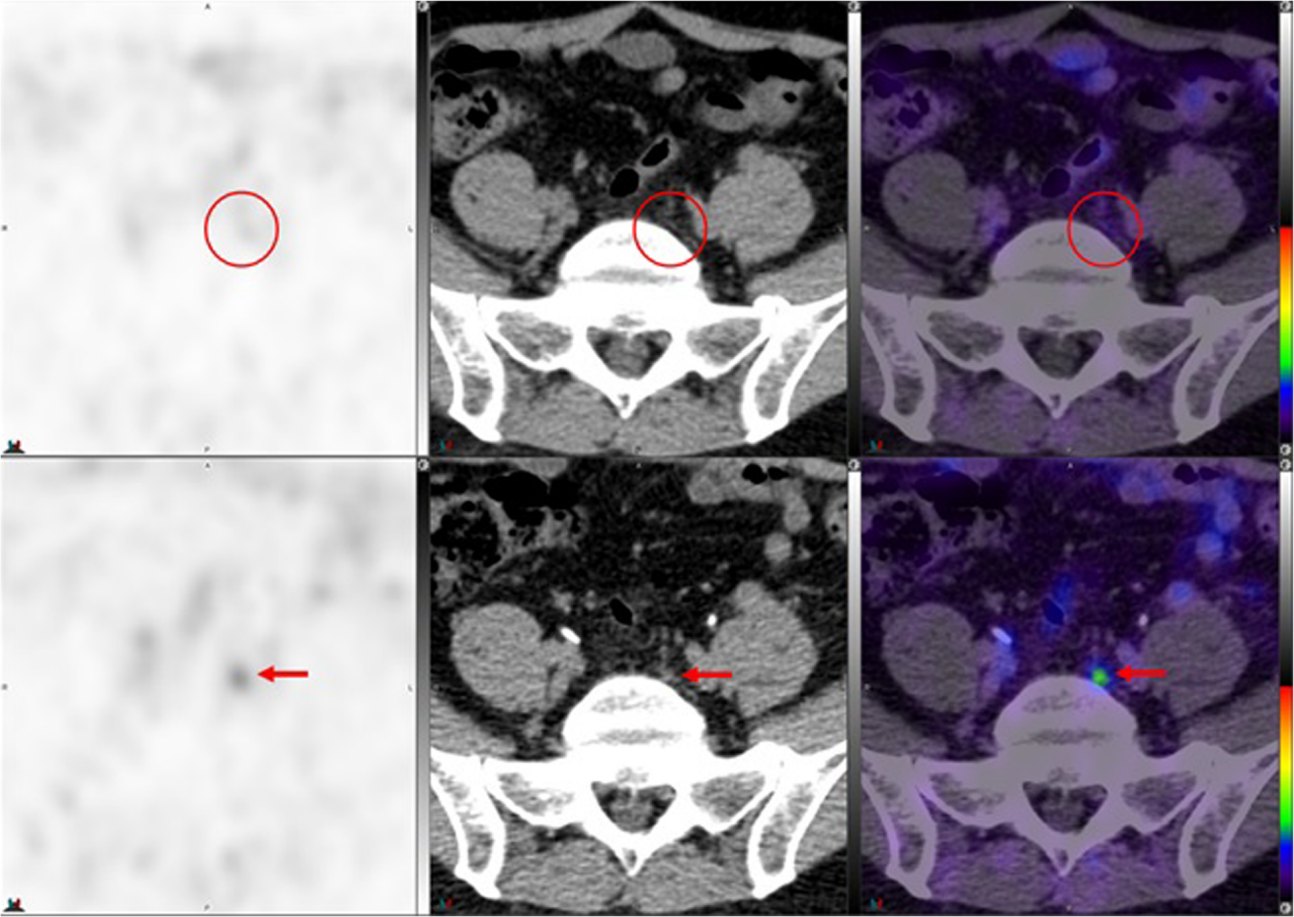
Top panel ^68^Ga-PSMA-11: axial PET, axial CT and axial fused PET/CT. Lower panel ^18^F –DCFPyL: axial PET, axial CT and axial fused PET/CT. Left sacral ganglion uptake with ^18^F –DCFPyL (SUVmax = 3.1) (arrows) but not with ^68^Ga-PSMA-11 (circles). By CT, similar to lumbar ganglia, there are typically no anatomic structures detectable in the region of paravertebral ganglia; however, location, bilaterality and multiplicity would provide clues. Prevertebral sacral ganglion, however, may appear band like, linear or curvilinear, and given the proximity to primary sites, it is probably the most challenging ganglia to differentiate from nodal metastasis. This is particularly true if uptake in the ganglia is similar to that of metastatic lymph nodes [25]

To date, there is no published systematic evaluation for lumbar ganglia uptake in prostate cancer patients imaged with ^68^Ga -PSMA-11. This could be due to minimal uptake, small size, and /or relatively limited image resolution. Werner et al. reported that the detection rate with ^18^F –DCFPyL uptake in lumbar ganglia was 72.4% [11].. Same study reported a gradually increasing ^18^F –DCFPyL uptake from L1 to L5. Given the paravertebral location, identification of PSMA uptake in sacral and lumbar region is relatively easy and should not be confused with metastatic lymph nodes. Even in the presence of misregistration artifacts, bilaterality and multiplicity of the uptake would provide clues to the benign nature of such ganglion uptake. Of importance, Werner et al. reported ^18^F –DCFPyL uptake in a paravertebral sacral ganglia was noted in 6.6% of patients. However, Rischpler et al. more recently reported ^68^Ga -PSMA-11 uptake in sacral ganglia to be much higher (46%) and localized in the prevertebral pelvic cavity [10]. Of importance, there is significant overlap in SUVmax values and even morphology between metastatic lymph nodes and ganglia [22, 23]. For pre-sacral and coeliac ganglia, even increasing SUVmax at delayed imaging does not reliably differentiates between ganglia and prostatic cancer lesion [24]. Therefore, of all the metabolically active ganglia in the body, due to the proximity to the primary site, prevertebral sacral ganglion is probably the most critical potential source of false positive that may mimic regional nodal metastases. Representative cases of lumbar and sacral (pre and paravertebral) ganglia uptake detected with ^68^Ga -PSMA-11 and ^18^F -DCFPyL radiotracers in same patients are provided in Figs. 10 and 11 Interestingly, we noticed sacral ganglia were less apparent on PSMA-11 compared to the newer and higher affinity PSMA agents.

## Discussion and conclusions

Our study had some limitations. SUV values were reported for reference and may be subject to error in measurements. Also, imaging acquisition and protocol were not standardized in our study. Differences in uptake duration, camera selection, number of iterations and subsets for OSEM may have contributed, at least in part, to the differences in the conspicuity of ganglia. Whilst sympathetic ganglia blocks are used in treating various pain disorders and PSMA uptake in different ganglia is frequently encountered, there is still a gap in the literature regarding the clinical significance of such uptake in the settings of prostate cancer diagnosis and or treatment. In our experience, we observed different patterns of ganglion visualization with ^18^F-DCFPyL compared to ^68^Ga-PSMA-11 which may result in dissimilar interpretative pitfalls. Reading physicians should be aware of the frequently encountered and occasionally different physiologic uptake of ^68^Ga-PSMA-11 and ^18^F DCFPyL in different ganglia. This is particularly important in patients imaged sequentially with different tracers to ensure that different ganglia uptake patterns should not be confused with new lesions. There are also parasympathetic ganglia in the head and neck region and pelvis. The former may not be visualized because of proximity to salivary and lacrimal glands and the latter due to proximity to the bladder but mixed populations of neural cells in various ganglia may account for differential uptake.

## Data Availability

Not applicable.

## Abbreviations

^68^Ga PSMA-11: Gallium-68 Prostate Specific Membrane Antigen-11
PET/CT: Positron Emission Tomography with Computerized Tomography
^18^F DCFPyL: 2-(3-(1-carboxy-5-[(6-[^18^F]fluoro-pyridine-3-carbonyl)-amino]-pentyl)-ureido)-pentanedioic acid
PC: Prostate Cancer
GMP: Good Manufacturing Practice
HBED: Glu-NH-CO-NH-Lys-(Ahx)
NDA: New Drug Application
FDA: Food and Drug Administration
CT-U: CT Urography
eGFR: Estimated Glomerular Filtration Rate
OSEM: Ordered Subset Expectation Maximization
MBq/kg: Megabecquerel/Kilogram
FWHM: Full-Width at Half-Maximum
ANS: Autonomic Nervous System
SUVmax: Maximum Standard Uptake Value
PTSD: Post-Traumatic Stress Disorder
